# Shock and Its Treatment*Read before the Association of Central Railroad Surgeons at Savannah Ga., March 7, 1893.

**Published:** 1893-04

**Authors:** J. I. Darby

**Affiliations:** Americus, Ga.


### Southern Medical Record.

A MONTHLY JOURNAL OF MEDICINE AND SURGERY.

VOL. XXIII. ATLANTA, GA., APRIL, 1893. No. 4.

### Original Articles.

### SHOCK AND ITS TREATMENT.*

BY J. I. DARBY, M. D., AMERICUS, GA.

Railway surgeons are, perhaps, called upon more frequently to treat individuals suffering from shock of injuries, than any other class of surgeons engaged in civil practice, and it therefore becomes necessary for them to have clear and comprehensive ideas of this condition, for without a proper knowledge of the cause, seat of lesion, and pathology, there cannot be any clear and rational course of treatment instituted in their efforts to relieve those thus injured. We know of no condition in which it can be more truthfully said, that the patient's life hangs by a thread, as it were, than in an extreme degree of shock, for it not infrequently happens that individuals thus injured, die under the treatment of able medical men, when there are no traumatisms in any part of the body of sufficient gravity so far as can be found upon post mortem examination, to produce the result, nor do we know of a condition in which a little wrong or misdirected treatment might be of more serious consequence to the unfortunate individual, therefore, it is a subject which should be studied well and properly understood by those whose duty it may be to treat this class of sufferers.

*Read before the Association of Central Railroad Surgeons at Savannah, Ga., March 7, 1893.

Experimental physiology has done much to demonstrate the pathology of shock and has clearly shown that the seat of lesion is mainly in the vasomotor centers of the nervous system, and that the coats of the blood vessels under the control of these centers lose temporarily their contractile power and the blood currents much of their velocity in consequence of this temporary paralysis, and that the heart is not suffering from an over supply of blood as was, formerly supposed. Shock has been defined to mean a sudden or instantaneous depression of the vital powers, with more or less purturbation of body and mind occasioned by injuries to the vasomotor centers or overwhelming moral calamity. The condition may exist in various degrees, and may be so slight and transitory as to be unnoticed or may be so profound as to cause instant death.

The varying degrees of depression of nerve force and of heart failure, which may be produced by the infliction of a wound, causes symptoms of shock to vary from momentary palor and mental confusion to a condition of profound prostration.

Ashhurst says: "The symptoms of an extreme degree of shock resemble those of approaching death very closely, for in this condition the patient may lie perfectly quiet, giving no heed whatever to anything that goes on around, with limbs helpless and prostrate as they may be placed, or as the chance of the moment may dictate; conscious, yet seeing only in a mist, and hearing none but loud and repeated questions, though a few rare instances are said to be met with in which the senses are exceedingly acute, with no paralysis, yet replying with difficulty in a syllabic, scarcely audible voice, and only executing with painful slowness some simple movements, often left half finished. The expression of the face is greatly changed. All the features, especially the nose, are smaller and shriveled, the weary eyes lose their lustre, and lie rolled upwards in deeply sunken sockets, surrounded by a dusky ring, the the pupils are usually dilated, and react very slowly to the stimulous of light. The skin and such parts of the mucous membranes as are visible are pale—the fingers and nails blue, and the skin on the palmer aspect frequently hanging in loose fields. Large drops of perspiration hang on the forehead and eye-brows. The whole body is cold, at times a shiver passes through all the limbs, and the loss of temperature is frequently so great as to make the thermometer fall two or more degrees. Common sensibility, and the sense of pain are usually much blunted over the whole body, only some paroxysm more sharp than ordinary can rouse to any movement, while the worst of news may be heard without emotion, so great is the already existing depression. The pulse is almost imperceptible, irregular, unequal, and very rapid, the arteries are small and the tension low. The respirations are very irregular, abnormally deep, sighing inspirations breaking suddenly into a series of very superficial ones, which are scarcely audible. Retention of urine occurs perhaps more frequently than not, and sometimes with this, there is partial suppression."

Now the above symptoms do not always manifest themselvs, even in fatal cases, but the patient will sometimes exhibit the most uncontrollable restlessness and excitability, often shouting again and again, at some imaginary being, or giving his whole attention to his condition, so much so, that it is impossible to get his attention upon anything else.

Injuries received in railroad wrecks are generally attended with more shock than similar injuries received otherwise, on account of the great panic and mental distress incident to accidents of this character, and the often prolonged exposure to weather, which is frequently cold and chilly, and the delayed medical attention in localities where doctors are procured with difficulty. Injuries of the brain and other internal organs are always attended with greater shock than similar lesions, on the external portions of the body, slight wounds of the intestines frequently resulting in death, even in cases where the most prompt and intelligent treatment is resorted to. When called to treat a case of shock, the surgeons' first duty is to place the patient in the recumbent posture, and loose any constricting wearing apparel, which might hinder the patient's circulation and respiration, and then proceed at once to use such remedies as would tend to re-establish the circulation and nervous sensibilities. This can, in a majority of instances, be done by the judicious use of cardiac stimulants and external warmth. My opinion is, that good whisky or brandy judiciously given when the patient is in a condition to swallow a sufficient amount of it, are among the best remedies we have with which to produce reaction, but as the surgeon does not always have these remedies with him, and the condition of the patient is frequently such as to preclude the use of stimulants thus administered, morphine and atropine may be used hypodermatically, with much reasonable hope of success. Strychnine and digitalis has been used by many eminent surgeons, and deservedly occupies a high place in the estimation of the profession. Ammonia and alcohol are frequently injected subcutaneously. Enemas of whisky and hot water are frequently injected into the rectum with decided benefit. The hypodermic use of nitroglycerine, it is said, has relieved patients when other reliable remedies had failed. External warmth applied in the form of hot rocks, jugs of hot water, and hot blankets, together with cloths wrung out of water as hot as the patient can bear, and applied over the region of the heart, have proven themselves to be remedies of greatest value. Friction with the hand, and rough towels, together with the liberal use of mustard, in suitable cases, should be resorted to, but in cases of severe scalds the friction and mustard should be omitted, especially over the parts thus injured. While I regard morphine and atropine as remedies of great value in the treatment of shock, I believe them to be very dangerous remedies in the hands of ignorant or careless doctors, for we know that an overdose of either of these drugs, would have the effect to deepen the already profound shock, therefore, it is always proper to wait a sufficient length of time for the first hypodermic of morphine and atrophine to be absorbed before repeating it, and if the morphine has to be repeated several times, the atrophine should be left out usually after the second dose. The patient should be kept in the recumbent position until the circulation is fully restored to its normal condition, or sufficiently so to prevent danger from heart failure. We should not lose sight of the fact, that it is possible for us to carry our efforts too far in trying to relieve this class of patients and produce with our stimulative remedies, a dangerous degree of reaction, but we should be exceedingly careful to carry our efforts in this direction only sufficiently far to make the individual's condition safe. Only a few years ago surgeons resorted to the lancet in the treatment of shock, believing that the heart was laboring under an over-supply of blood which was embarrassing its functions, but in more recent years, since we have a more intelligent comprehension of the true pathology of this condition, and it has been satisfactorily demonstrated that the heart is partially paralyzed through injuries in the vasomotor centers and not an over burden of blood, the lancet has been laid to rest and only called into requisition in the reactive stage if at all.

If the reaction from shock is too great, and the patient suffers from inflammation threatenings, antipyretic remedies should be promptly resorted to, and the bowels kept open with saline cathartics, and the patient fed sparingly on unstimulating diet, until all active febrile movement has abated.

We sometimes find ourselves called upon to treat emergency cases, where it is necessary to perform operations during the existence of shock—for instance when important blood vessels are wounded and bleeding dangerously—when the abdominal or thoracic walls are opened, or in case of great injuries to the skull, in which pieces of depressed bone are threatening fatal results. Sometimes it is necessary to amputate a mangled limb, or reduce a dislocation, or perform a herniotomy, while the patient is suffering from shock, but it is far better that operations of a capital nature, should not be performed under these conditions, if they can with any degree of safety be deferred until reaction is at least partially established, and in those cases which cannot be safely postponed, and an anæsthetic required, ether should be used instead of chloroform, as it stimulates the heart and lessens the danger of fatal depression. These patients should, as far as possible, have the benefit of a bloodless operation, and such remedies as are necessary, to prevent further shock. Hemorrhage associated with shock is often a dangerous complication, and should be treated promptly, as the loss of a very shall amount of blood under these circumstances might result fatally, but cases which are promptly treated might recover. When shock results mainly from excessive loss of blood, and the heart for want of the proper amount of circulating fluid, is failing to properly perform its functions, we should at once proceed to supply the necessary amount, by injecting into the circulation, through the femoral, or other large artery, a sufficent quantity of warm saline solution, to make up for the lost blood and thereby, not only afford temporary benefit by mechanical means, but also permanently benefiting our patient, by placing in his vessels a substance, a portion of which good mother nature utilizes in building up his impoverished blood. If this can be done before the shock becomes too profound, it is wonderful how great a loss of blood may be recovered from. Dr. R. H. M. Dawborn, of New York City, says that of all the substances so far resorted to for this purpose, chloride of sodium and hot water have proven themselves to be of greatest utility and value. The proportions of salt and water, are a heaped teaspoonful of the former, to a quart of the latter, taking care to boil and strain the water before using it, the hot water should never be used without the salt, for an omission of this might result disastrously to the patient, if much of the solution was used. The only instruments needed to inject the solution, is a hypodermic needle, a Davidson's or other good soft rubber syringe and a piece of soft Neiaton catheter with its point cut off, so as to fit over the needle. We then find a superficial artery by its pulsations, preferably the femoral, below poupart ligament, and we next take the hypodermic needle, not as yet attached to the catheter and push it directly into the artery, going slowly until the bright arterial blood is seen welling up in the needle, the artery here being of large size, the needle can hardly miss it when in the hands of a careful surgeon. When the little thread like stream of blood is seen spinning up through the needle, we then slip the catheter over the base or large part of the needle and wrap a strong thread several times around it, to prevent leakage when the fluid is forced in by the syringe. We next emerse the syringe in the basin of saline fluid, and after thoroughly exhausting all of the air from the syringe, by filling it with the solution and holding the pipe upwards until the fluid comes over, pass the pipe of it into the open end of piece of catheter proceed to pump the fluid through the needle into the artery, remembering that the process is necessarily a slow one and that it will probably require a half hour to pump in a pint of the solution, which is the amount ordinarily used, though in some cases it might be justifiable to use a quart or even more. If the surgeon does not feel justified in penetrating the coats of the artery, with the hypodermic needle, he may inject the warm solution into the rectum, and connective cellular tissues, and get much of it absolved to the great benefit of his patient. The temperature of the solution must not be lost sight of as it is quite dangerous to inject into the blood currents fluids the temperature of which are below the normal temperature of the human body. It should be at least 100 degrees F. and may be used much warmer if desired.

